# Ezetimibe ameliorates clinical symptoms in a mouse model of ankylosing spondylitis associated with suppression of Th17 differentiation

**DOI:** 10.3389/fimmu.2022.922531

**Published:** 2022-08-17

**Authors:** Jeonghyeon Moon, Seon-Yeong Lee, Hyun Sik Na, A Ram Lee, Keun-Hyung Cho, Jeong Won Choi, Sung-Hwan Park, Mi-La Cho

**Affiliations:** ^1^ Departments of Neurology and Immunobiology, Yale School of Medicine, New Haven, CT, United States; ^2^ Rheumatism Research Center, Catholic Research Institute of Medical Science, College of Medicine, The Catholic University of Korea, Seoul, South Korea; ^3^ Lab of Translational ImmunoMedicine, Catholic Research Institute of Medical Science, College of Medicine, The Catholic University of Korea, Seoul, South Korea; ^4^ Department of Biomedicine & Health Sciences, College of Medicine, The Catholic University of Korea, Seoul, South Korea; ^5^ Division of Rheumatology, Department of Internal Medicine, Seoul St. Mary's Hospital, College of Medicine, The Catholic University of Korea, Seoul, South Korea; ^6^ Department of Medical Life Sciences, College of Medicine, The Catholic University of Korea, Seoul, South Korea

**Keywords:** ankylosing spondylitis (AS), ezetimibe, helper T cell 17, drug repositioning, autoimmune disease (AD)

## Abstract

Ankylosing spondylitis (AS) is a chronic inflammatory disease that causes spinal inflammation and fusion. Although the cause of AS is unknown, genetic factors (e.g., HLA-B27) and environmental factors (e.g., sex, age, and infection) increase the risk of AS. Current treatments for AS are to improve symptoms and suppress disease progression. There is no way to completely cure it. High blood cholesterol and lipid levels aggravate the symptoms of autoimmune diseases. We applied hyperlipidemia drugs ezetimibe and rosuvastatin to AS mice and to PBMCs from AS patients. Ezetimibe and rosuvastatin was administered for 11 weeks to AS model mice on the SKG background. Then, the tissues and cells of mice were performed using flow cytometry, computed tomography, immunohistochemistry, and immunofluorescence. Also, the normal mouse splenocytes were cultured in Th17 differentiation conditions for *in vitro* analysis such as flow cytometry, ELISA and RNA sequencing. The 10 AS patients’ PBMCs were treated with ezetimibe and rosuvastatin. The patients’ PBMC were analyzed by flow cytometry and ELISA for investigation of immune cell type modification. Ezetimibe caused substantial inhibition for AS. The present study showed that ezetimibe inhibits Th17 cell function, thereby slowing the progression of AS. It is well known that statins are more effective in reducing blood lipid concentrations than ezetimibe, however, our results that ezetimibe had a better anti-inflammatory effect than rosuvastatin in AS. This data suggests that ezetimibe has an independent anti-inflammatory effect independent of blood lipid reduction. To investigate whether ezetimibe has its anti-inflammatory effect through which signaling pathway, various *in vitro* experiments and RNA sequencing have proceeded. Here, this study suggests that ezetimibe can be an effective treatment for AS patients by inhibiting Th17 differentiation-related genes such as IL-23R and IL-1R. Thus, this study suggests that ezetimibe has therapeutic potential for AS through inhibition of Th17 differentiation and the production of pro-inflammatory cytokines.

## Introduction

Ankylosing spondylitis (AS) is a chronic autoimmune disease that involves prolonged inflammation of the spinal joints (1–3). Symptoms of AS occur before the age of 40 years (4). Initial symptoms of AS are chronic obtuse pain in the posterior waist or gluteal region, combined with stiffness of the lower back. AS progression can trigger ossification of the lumbosacral joint and the vertebrae (5–8). In addition, several organ systems may be affected, such as the eyes (9), intestine (10), skin (11), and heart (12). Although the underlying mechanism of AS development *via* autoimmune or autoinflammation is partially known, the accurate cause of AS is still unknown (13–15). AS is influenced by both genetic and environmental factors (16). More than 90% of AS patients have human leukocyte antigen (HLA)-B27 (17, 18). Immune cells (e.g., Th17 cells) and proinflammatory cytokines (e.g., IL-6, IL-17, and IL-1β) are important in the pathogenesis of AS (19). The inhibition of tumor necrosis factor-alpha (TNF-α) can relieve the symptoms of AS (20–22). The pathogenesis of AS is influenced by environmental factors including sex, age, smoking, infection, toxins, and pollutants (23). Some previous studies have shown that hyperlipidemia appears to be related to the severity of AS (24–26). For this reason, the results of this study are expected to alleviate AS symptoms when lipid levels are lowered in AS animal models and patient cells.

Statins and ezetimibe are commonly used for the treatment of hyperlipidemia (27). Statins, also known as HMG-CoA reductase inhibitors, are the most common cholesterol-lowering drugs (28). Statins act on the liver to inhibit lipid synthesis and HMG-CoA reductase activity (29).

Contrary to statin, ezetimibe reduces the blood cholesterol level by preventing cholesterol absorption in the small intestine. Ezetimibe is a Food and Drug Administration-approved lipid-reducing drug that blocks the intestinal cholesterol transporter Niemann-Pick C1-Like 1 (NPC1L1) (30, 31). Ezetimibe alone or in combination with a statin can reduce the blood low-density lipoprotein level in patients with hyperlipidemia (32–34). Although ezetimibe is less effective than statins for reducing blood lipid concentrations, it has fewer serious side effects such as muscle damage, increased risk of diabetes mellitus, and abnormal blood levels of liver enzymes (28). Furthermore, ezetimibe exerts an anti-inflammatory effect in inflammatory diseases, such as rheumatoid arthritis and atherosclerosis (35, 36). Although ezetimibe elicits an anti-inflammatory response by suppressing nuclear factor kappa-light-chain-enhancer of activated B cells (NF-κB) transcription through the mitogen-activated protein kinase pathway in macrophages, the molecular mechanism underlying the anti-inflammatory effect of ezetimibe is unclear (37, 38). Hence, we investigated whether the ezetimibe could ameliorate AS in a mouse model through an anti-inflammatory effect.

Th17 cells have roles in multiple human autoimmune diseases, including AS (39). The number of Th17 cells and the serum levels of Th17-related cytokines (e.g., IL-17 and TNF-α) are significantly elevated in autoimmune disease (40–42). In addition, regulatory T (Treg) cells are implicated in self-tolerance; they are involved in cancer (41) and autoimmunity (43). Treg and Th17 cells have adversarial functions in autoimmune diseases. Therefore, the Th17/Treg balance is crucial for the treatment of cancer and autoimmunity (44). The ratio of Th17/Treg cells is increased in AS patients; its modulation can ameliorate symptoms (45, 46).

Oxidized low-density lipoprotein influences the Th17/Treg balance. A high serum concentration of oxidized low-density lipoprotein is negatively correlated with the number of Treg cells and positively correlated with the number of Th17 cells (47). Therefore, we expected that the reduction of blood lipid levels using ezetimibe and statin would ameliorate AS disease by reducing the number of Th17 cells.

Ezetimibe, an inhibitor of oxidized low-density lipoprotein, ameliorated AS in mice by modulating the Th17/Treg cell balance. Ezetimibe and a statin improved AS exacerbations in a mouse model. Notably, ezetimibe, which has a weaker blood lipid-lowering effect than statins, showed a greater regulatory effect on the inflammation than did the statin (48, 49). These data were inconsistent with our initial hypothesis. In AS, the results that ezetimibe had a better anti-inflammatory effect than rosuvastatin, which strongly lowers blood lipid concentration by blocking lipid synthesis in the liver, suggests that ezetimibe has an independent anti-inflammatory effect independent of blood lipid reduction. To investigate whether ezetimibe has its anti-inflammatory effect through which signaling pathway, various *in vitro* experiments and RNA sequencing have proceeded. Here, this study suggests that ezetimibe can be an effective treatment for AS patients by inhibiting Th17 differentiation-related genes such as IL-23R and IL-1R.

## Materials and methods

### Mice

BALB/c background SKG mice were kindly provided by Professor Shimon Sakaguchi (Department of Experimental Immunology, World Premier International Immunology Frontier Research Center, Osaka University) (50). Mice were maintained under specific-pathogen-free conditions and fed standard mouse chow (Ralston Purina, St. Louis, MO, USA) and water ad libitum. The experiments were assessed and approved by the Institutional Animal Care and Use Committee of the School of Medicine and the Animal Research Ethics Committee of the Catholic University of Korea (CUCM-2015-0063-01); they were conducted in accordance with the Laboratory Animals Welfare Act, Guide for the Care and Use of Laboratory Animals.

### Spondyloarthritis induction and ezetimibe treatment

Microbial β-1,3-glucan (curdlan) (3 mg/kg) was intraperitoneally injected into 8–10-week-old SKG mice. Ezetimibe (10 mg/kg) was administered orally daily to SpA-induced SKG mice for 6 weeks. Clinical scores were measured weekly for 6 weeks: 0 = no swelling or redness, 0.1 = swelling or redness of the digits, 0.5 = mild swelling and/or redness of the wrist or ankle joints, and 1 = severe swelling of larger joints. Scores of affected joints were summed for each mouse.

### *In vivo* micro-computed tomography

Micro-computed tomography imaging was performed using a bench-top cone-beam-type *in vivo* animal scanner (mCT 35; Scanco Medical, Brüttisellen, Switzerland). Mice were euthanized; their spines were dissected and immediately placed in 10% neutral buffered formalin, where they were incubated for 2 weeks. Samples were imaged at settings of 60 kVp and 166 μA using a 0.25-mm-thick aluminum filter. The pixel size was 16 μm and the rotation step was 0.6°. Cross-sectional images were reconstructed using a filtered back-projection algorithm (NRecon software; Bruker microCT, Kontich, Belgium). For each scan, a stack of 286 cross-sections was reconstructed at 2,000 × 1,335 pixels.

### Histological analysis

Ten percent neutral-buffered formalin-fixed tissue samples from peripheral joints, spine, and small intestine were embedded in paraffin; sections were produced at a thickness of 7 µm. The tissue sections were dewaxed using xylene, then dehydrated in an alcohol gradient. The samples were stained with hematoxylin and eosin; stained sections of intestinal tissue were scored for inflammation. The histological score of intestinal tissue was determined on a scale of 1-4: 1 = few infiltrating cells, 2 = mild infiltration of the intestine (0–30% of the mucosa), 3 = inflammation of intestine (30–70% of the mucosa), and 4 = inflammation in > 70% of the intestinal mucosa (51–53).

### Immunohistochemistry

Immunohistochemical analysis was performed using the Vectastain ABC Kit (Vector Laboratories, Burlingame, CA, USA). Tissues were first incubated overnight at 4°C with primary antibodies against IL-17, IL-10, and IL-1β (Santa Cruz Biotechnology, Santa Cruz, CA, USA); they were then incubated with biotinylated secondary antibodies against goat (Santa Cruz Biotechnology) and streptavidin-peroxidase complex (Vector Laboratories) for 1 h. Colored products were developed using the chromogen 3,3-diaminobenzidine (Dako, Carpinteria, CA, USA). Histological assessments were performed by two independent, blinded observers. Images were captured using a DP71 digital camera (Olympus, Center Valley, PA, USA) attached to a BX41 microscope (Olympus) at 3400× magnification.

### Confocal microscopy

Spleen tissues were obtained from SKG mice in which AS had been induced by curdlan injection at 6 weeks of age. To identify populations of Th17 and Treg cells, the tissues were reacted with fluorescein isothiocyanate-conjugated anti-CD4, allophycocyanin-conjugated anti-IL-17, allophycocyanin-conjugated anti-CD25, phycoerythrin-conjugated anti-FOXP3, and Alexa Fluor 405-conjugated anti-CCR9 antibodies (eBioscience, San Diego, CA, USA). Stained tissue sections were observed under a confocal microscope (LSM 510Meta; Carl Zeiss, Oberkochen, Germany). Stained cells were enumerated using Pannoramic MIDI and Pannoramic viewer (3D Histech Ltd., Hungary).

### Murine T-cell isolation and Th17 differentiation

Mouse spleen cells were cultured in RPMI 1640 medium with 5% fetal bovine serum. CD4-positive T cells were stimulated with plate-bound anti-CD3 (0.5 μg mL^−1^), soluble anti-CD28 (1 μg mL^−1^; BD Biosciences), anti-interferon-γ (2 μg mL^−1^), and anti-IL-4 (2 μg mL^−1^) antibodies, as well as recombinant TGF-β (2 ng mL^−1^) and recombinant IL-6 (20 ng mL^−1^) (R&D Systems), for 3 days. Next, drug-treated splenocytes were cultured with 10 and 20 μM ezetimibe at 37°C for 3 days.

### Enzyme-linked immunosorbent assay

The quantities of IL-17, IL-10, IFNγ, and TNF-α in culture supernatants were quantified by sandwich enzyme-linked immunosorbent assay (ELISA; R&D Systems). Alkaline phosphate (Sigma-Aldrich) was employed for signal development. Absorbance at 405 nm was measured using an ELISA microplate reader (Molecular Devices).

### Flow cytometry

To evaluate CD4+IL-17+ (Th17), CD4+IFN-γ+ (Th1), CD4+IL-4+ (Th2), CD4+CD25+Foxp3+ (Treg), and CD3-CD45+RORγt+CD19- (ILC3) cells *ex vivo*, cell pellets were obtained from spleen tissues that had been isolated from ezetimibe- and vehicle-treated AS SKG mice (all antibodies from eBioscience). Spleen cells from wild-type BALB/c mice were used for *in vitro* analyses of Th1, Th2, Th17, Treg, and ILC cells. The PBMC of AS patients were stained using anti- human CD4, IL-17, CD25 and Foxp3 fluorescence conjugated antibodies (all antibodies from eBioscience).

### RNA-sequencing

Using TRIzol reagent, mRNA was isolated from naïve T cells, differentiated Th17 cells, and cells that had been treated with ezetimibe and rosuvastatin under Th17 differentiation conditions. RNA sequencing analysis was performed *via* next-generation sequencing. Expression data were preprocessed using the range migration algorithm, then subjected to quantile normalization. The Kyoto Encyclopedia of Genes and Genomics (KEGG) pathway was used to represent the molecular interaction and expression of Th17 differentiation related gene pathway.

### Patients and ethical statement

A total of 10 patients were prospectively enrolled from a single LT clinic at Seoul St. Mary Hospital between January 2019 and August 2020. Samples of 6 out of 10 patients had sufficient cell numbers (>2*10^6 cells) and were used for both Th17 and Treg investigations. The remaining 4 patients did not have sufficient cell numbers, they were used only for Th17 experiments. This study was approved by the institutional review board of Seoul St. Mary’s Hospital (KC17TNSI0237) and performed in accordance with the Declaration of Helsinki.

### Ezetimibe and rosuvastatin treatment to patient’s PBMC

A total of 10 AS patients’ blood was collected. PBMCs were isolated from this blood using Ficoll-Paque PLUS (GE Healthcare). Each cell was seeded in a 12-well dish at 5*10^5 and cultured. PBMC were incubated for one day with the non-treated, the 20 μM ezetimibe, and the 20 μM rosuvastatin. Four patients had insufficient PBMCs and were used only for Th17 confirmation trials. Then the cells were stimulated for 4 h with phorbol 12-myristate 13-acetate (PMA) (25 ng/mL) and ionomycin (250 ng/mL) in the presence of GolgiStop™ (BD Biosciences),. The culture supernatant were collected for ELISA analysis and cells were collected for flow cytometry.

### Statistical analysis

Data are presented as means ± standard errors of the mean. Statistical analyses were performed using SPSS 20.0 for Windows (IBM Corp., Armonk, NY, USA). Differences between two groups were analyzed using the Mann–Whitney U test under the assumption of equal variance. A value of *p <* 0.05 was considered indicative of statistical significance.

## Results

### Ezetimibe inhibits AS progression in a mouse model

**Figure 1:**
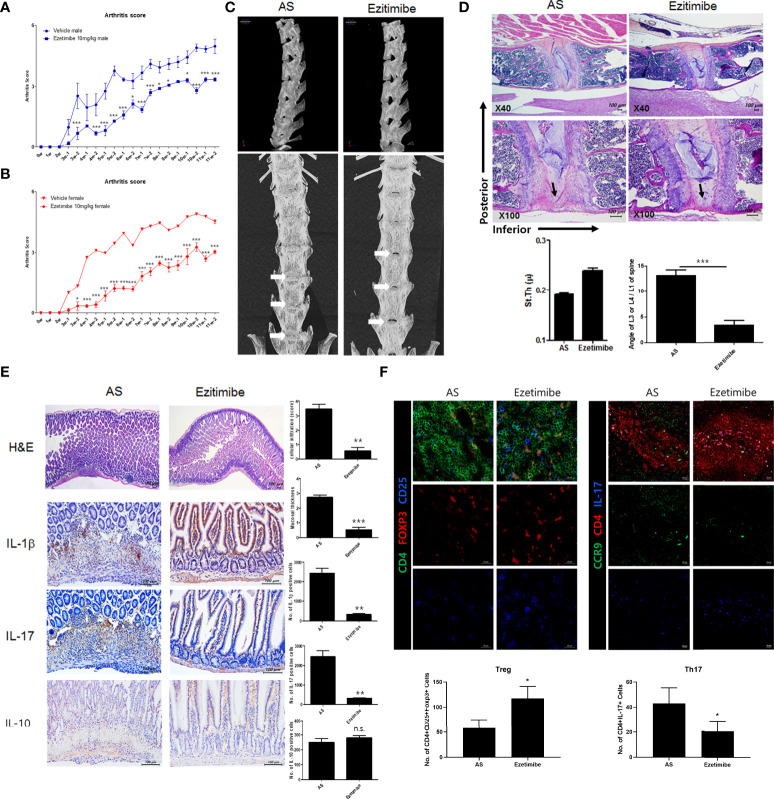
Ezetimibe ameliorated AS symptoms in mice. **(A, B)** Arthritis score and incidence in ezetimibe-treated and untreated AS mice. **(C)** Computed tomography showed that ezetimibe has a therapeutic effect on the spine in AS mice. Osteogenesis was observed in the lumbar disc region of AS mice (white arrows). **(D)** Immunohistochemistry images showed that the anterior lumbar spine of untreated AS mice exhibited stenosis and osteogenesis (black arrows). Scale bars = 100 µm. **(E)** Immunohistochemistry of small intestine tissue showed that ezetimibe decreased mucosal wall thickness, cell infiltration, and expression levels of proinflammatory cytokines (e.g., IL-1β and IL-17). Scale bars = 100 µm. **(F)** Immunofluorescence showed that ezetimibe reduced Th17 cell number and increased Treg cell number. Scale bars = 20 µm. Values are means ± standard errors of the mean (SEMs) of three independent experiments. *p < 0.05, **p < 0.01, ***p < 0.001.

To investigate the effects of ezetimibe and rosuvastatin on AS progression, we treated AS mice with ezetimibe and rosuvastatin for 11 weeks. Both ezetimibe and rosuvastatin inhibited the progression of AS. However, the effect of ezetimibe was greater than the effect of rosuvastatin (data not shown). We evaluated foot swelling and redness to assess systemic joint inflammation. Ezetimibe significantly inhibited joint inflammation *in vivo* (**Figures 1A, B**). In the AS model, the spine was curved inward; however, ezetimibe prevented bending of the spine (**Figure 1C**). Immunohistochemistry showed that the anterior lumbar spine of AS mice was stenotic and exhibited osteogenesis with reduced fluidity. However, the spine of ezetimibe-treated mice maintained a thick disc and a normal curvature; lumbar bone stenosis did not occur (**Figure 1D**). Furthermore, ezetimibe inhibited the expression of proinflammatory cytokines in the small intestine. Cell infiltration, mucosal wall thickness, and proinflammatory cytokines (IL-1β and IL-17) were significantly decreased in ezetimibe-treated mice (**Figure 1E**). Additionally, the CD4+CD25+Foxp3+ cell count was increased and the CD4+IL-17+ cell count was decreased in ezetimibe-treated mice, according to immunofluorescence images (**Figure 1F**). Therefore, unlike rosuvastatin, which reduces blood lipid concentrations, ezetimibe inhibits AS progression independently of lipid regulation. Ezetimibe inhibits intestinal tissue damage caused by inflammatory cells.

### Ezetimibe regulates systemic immune cells by reducing IL-17 expression

**Figure 2:**
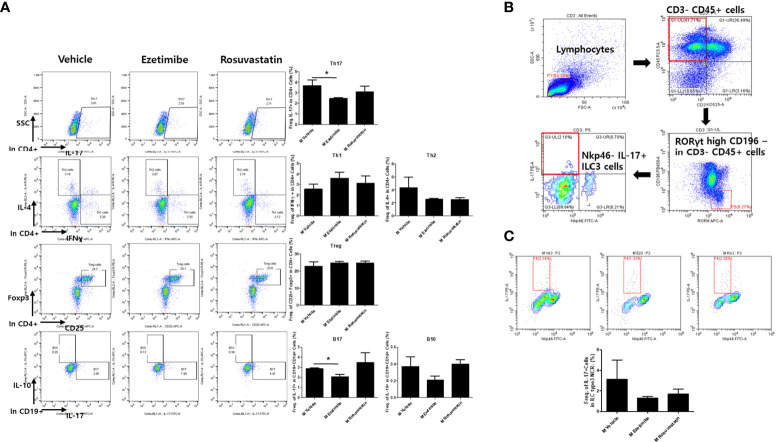
Role of ezetimibe in regulation of IL-17-producing immune cell populations in AS mice. **(A)** The numbers of CD4+IL-17+ (Th17), CD4+IFNγ+ (Th1), CD4+IL-4+ (Th2), CD4+CD25+Foxp3+ (Treg), CD1d+CD19+IL-17+ (B17), and CD1d+CD19+IL-10+ (B10) cells of mouse splenocytes were measured by *ex vivo* flow cytometry. **(B)** Gating strategy for IL-17-producing ILC3 cells. **(C)** IL-17+ ILCs in mouse splenocytes detected by flow cytometry. Values are means ± SEMs of three independent experiments. *p < 0.05.

We next investigated the mouse splenocyte population. Flow cytometry showed that the number of CD4+IL-17+ (Th17) cells was significantly decreased in ezetimibe-treated mice. However, no significant changes were observed in the numbers of other helper T-cell types, such as CD4+IFNγ+ (Th1), CD4+IL-4+ (Th2), and CD4+CD25+Foxp3+ (Treg) cells. Furthermore, the number of IL-17-producing CD1d+CD19+ cells decreased (**Figure 2A**). Since ezetimibe is known to act in small intestine cells, innate lymphoid cells (ILC) known to reside in small intestine cells were measured (**Figure 2B**). The quantity of IL-17 producing NKP46+ILC3, which is expressed by ILCs, tended to decrease in the ezetimibe-treated group (**Figure 2C**). Therefore, ezetimibe inhibits IL-17 production in immune cells.

### Effects of ezetimibe on cytokine expression in immune cells *in vitro*

**Figure 3:**
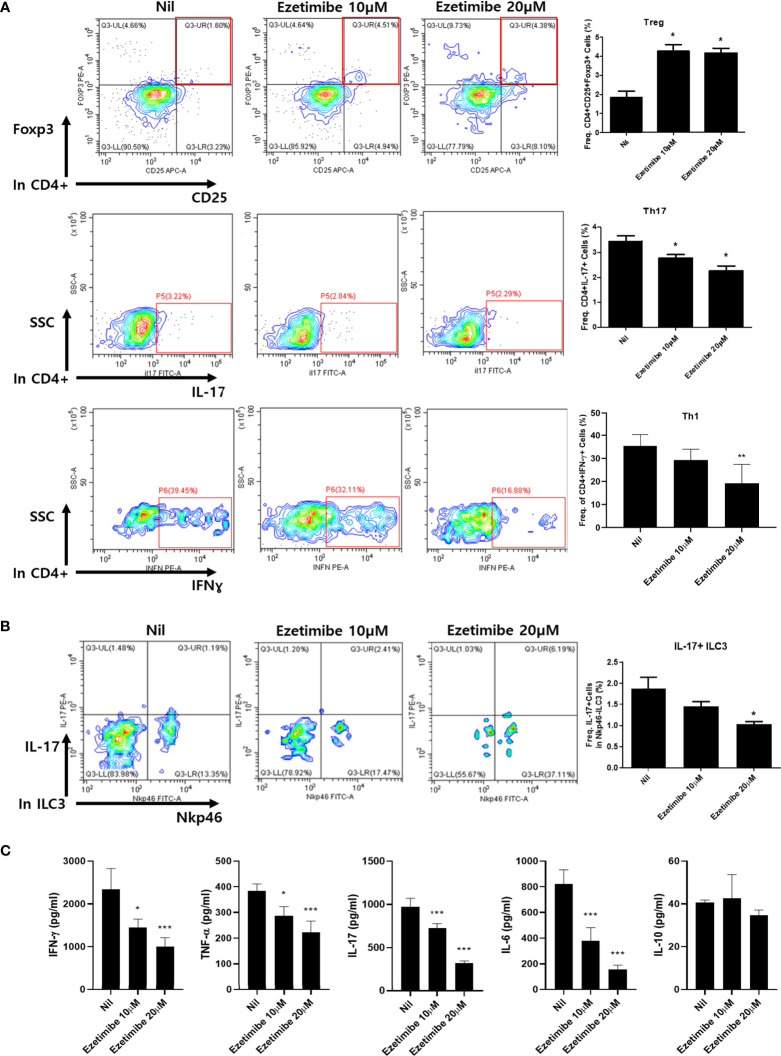
Effects of ezetimibe on mouse splenocytes *in vitro*. **(A)** The populations of Treg, Th17 and Th1 cells were analyzed by flow cytometry. **(B)** IL-17-producing ILC3 population. **(C)** The concentrations of IFN-γ, TNF-α, IL-17, IL-6 and IL-10 were measured by ELISA in culture supernatants. Values are means ± SEMs of three independent experiments. *p < 0.05, **p < 0.01, ***p < 0.001.

To determine whether ezetimibe affects immune cell differentiation, mouse splenocytes were cultured *in vitro*. Mouse splenocytes were placed on a 0.5 µg/mL anti-CD3ϵ coated dish for T-cell activation, then treated with 10 and 20 µM ezetimibe for 1 day. The numbers of Treg cells were significantly increased by treatment with 10 and 20 µM ezetimibe. In contrast, the numbers of Th17 and Th1 cells were reduced by ezetimibe (**Figure 3A**). The number of IL-17+ ILC3 cells was also reduced by 20 µM ezetimibe (**Figure 3B**). Additionally, the IFN-γ, TNF-α, IL-17 and IL-6 levels were substantially reduced by ezetimibe. However, the IL-10 level was unaffected (**Figure 3C**). Therefore, ezetimibe regulates T-cell expansion and proinflammatory cytokine secretion by immune cells.

### Effects of ezetimibe on expression patterns of differentiation-related genes in Th17 cells

**Figure 4:**
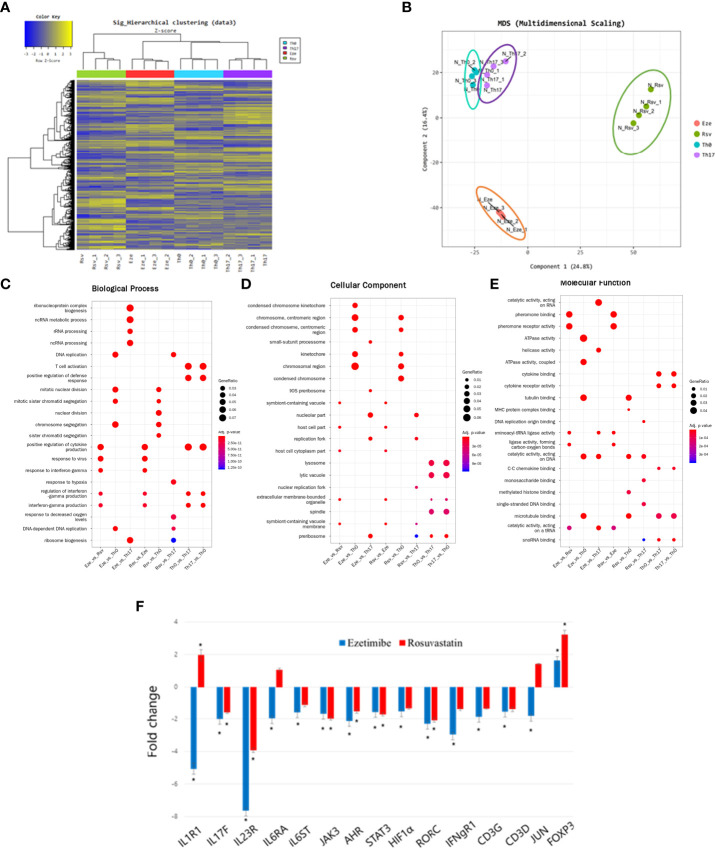
Ezetimibe inhibits Th17 cell differentiation. **(A)** Hierarchical clustering images and **(B)** multidimensional scaling images revealed the gene expression patterns of naïve T cells (Th0), differentiated Th17 cells (Th17), ezetimibe-treated Th17 cells (Eze), and rosuvastatin-treated Th17 cells (Rsv). **(C)** Biological process, **(D)** cellular component, and **(E)** molecular function images showing significant changes in gene expression. **(F)** Ezetimibe and rosuvastatin modulated the expression patterns of Th17 differentiation-related genes. Values are means ± SEMs of three independent experiments. *p < 0.05.

We hypothesized that ezetimibe has other effects on immune cells. Because ezetimibe inhibits Th17 differentiation, mouse splenocytes were treated with ezetimibe and rosuvastatin for 1 day under Th17 differentiation conditions. mRNA sequencing plus hierarchical clustering and multidimensional scaling images revealed significant changes in gene expression among naïve T cells (Th0), differentiated Th17 cells (Th17), ezetimibe-treated Th17 cells (Eze), and rosuvastatin-treated Th17 cells (Rsv) (**Figures 4A, B**). The gene expression patterns were compared in categories of biological process, cellular component, and molecular function. RNA processing-related gene expression of ezetimibe treated-Th17 cells exhibited significant changes, compared to untreated Th17 cells (**Figure 4C**). Ezetimibe treated-Th17 cells exhibited altered expression patterns of genes associated with chromosome regulation and catalytic activity, compared to non-treated Th17 cells and Th0 cells (**Figures 4D, E**). In addition, ezetimibe significantly modulated the expression patterns of Th17 differentiation-related genes. Increased expression levels were observed for the IL-1R1, IL-17F, IL-23R, IL-6RA, IL-6ST, JAK3, AHR, STAT3, HIF1α, RORC, IFNgR1, CD3G, CD3D, and JUN genes, all of which promote Th17 differentiation; the expression level of Foxp3, which inhibits Th17 differentiation, also significantly increased (**Figure 4F**). Therefore, ezetimibe inhibits Th17 cell differentiation.

### Ezetimibe reduces the number of Th17 cells in PBMCs from AS patients

**Figure 5:**
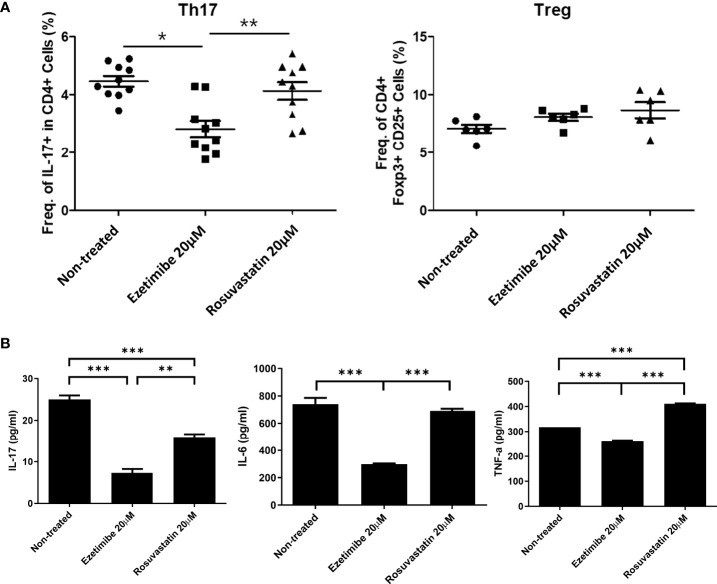
Effects of ezetimibe in PBMCs from AS patients. **(A)** Numbers of Th17 and Treg cells among PBMCs from AS patients, measured by flow cytometry. **(B)** Concentrations of IL-17, IL-6, and TNF-α in culture supernatants, measured by ELISA. Values are means ± SEMs of three independent experiments. *p < 0.05, **p < 0.01, ***p < 0.001.

To determine whether ezetimibe inhibits Th17-cell differentiation in AS patients, PBMCs from 10 AS patients were treated with 20μM of ezetimibe and 20μM rosuvastatin for 1 day respectively. Then, cells were collected for flow cytometry analysis and the culture supernatants were collected for the investigation of the concentration of pro-inflammatory cytokines using ELISA. Although ezetimibe and rosuvastatin treatment did not affect the Treg population, ezetimibe treatment significantly reduced the number of Th17 cells among AS patients’ PBMCs (**Figure 5A**). The IL-17 concentration was reduced in the ezetimibe and rosuvastatin treatment groups in AS patients’ PBMC culture supernatant. However, the levels of IL-6 and TNF-α were reduced only by ezetimibe treatment (**Figure 5B**). Therefore, ezetimibe inhibits the Th17 cell differentiation in AS patients’ systemic immune cells. Also it has an anti-inflammatory effect and prevents pro-inflammatory cytokines on immune cells in AS patients.

## Discussion

AS is a chronic inflammatory disease that predominantly affects men, begins in young adulthood, and results in immobility of the spine and sacroiliac joints (54). HLA-B27, an MHC-I surface protein encoded by the MHC B gene on human chromosome 6, predisposes carriers to AS (55). HLA-B27 presents peptide antigens to immune cells; it is associated with AS and associated inflammatory diseases (56). Therefore, AS patients are typically prescribed TNF-α inhibitors, but the complex pathogenesis of AS limits the efficacy of such treatment. In this study, we investigated a novel method that could be used in addition to treatments involving HLA-B27 regulation and TNF-α inhibitors.

We initially examined whether the reduction of blood lipid levels alleviates AS by administering ezetimibe and rosuvastatin, which reduce blood lipid concentrations *via* distinct mechanisms, for 11 weeks. Both ezetimibe and rosuvastatin decreased the clinical arthritis score in AS mice. However, ezetimibe-treated mice exhibited a greater reduction in arthritis score than did rosuvastatin-treated mice. Rosuvastatin is superior to ezetimibe in terms of reducing blood lipid levels (57). Therefore, these results imply that ezetimibe has other, as-yet-unknown inflammation-reducing and immune cell-modulating activities.

Ezetimibe also alleviated spinal stenosis, an important symptom of AS, by reducing the increased disc thickness characteristic of AS; this alleviated damage and reduced vertebral angle deformation, as well as the associated vertebral stenosis.

Lipids induce inflammation by increasing proinflammatory cytokine levels (58). Ezetimibe decreased the expression levels of proinflammatory cytokines in intestinal immune cells and enterocytes. Spleen tissue immunofluorescence staining and flow cytometry of mouse splenocytes showed that ezetimibe regulated intestinal inflammatory cells and inflammatory cytokines, as well as systemic immune cells.

Flow cytometry showed changes in systemic immune cells in AS mice and in normal mouse splenocyte populations cultured in the presence of anti-CD3 *in vitro*. Ezetimibe reduced the expression levels of IL-17 in B cells and ILCs, as well as T cells. Therefore, ezetimibe ameliorated inflammation and tissue damage by reducing IL-17 expression and suppressing Th17 differentiation.

To identify the pathway by which ezetimibe inhibits IL-17 expression and Th17 differentiation, we subjected to splenocytes to RNA-Seq. The results showed that ezetimibe and rosuvastatin inhibited Th17 differentiation by distinct mechanisms. Hierarchical clustering heatmap analysis showed contrasting gene expression patterns in ezetimibe-treated and untreated conditions under a Th17 cell differentiation-promoting environment.

Gene ontology data showed significantly increased expression levels of genes associated with T-cell activation and cytokine production by differentiated Th17 cells. These findings indicated the presence of normal Th17 differentiation. Furthermore, the expression levels of genes associated with RNA processing, including RNA metabolism and ribosome biogenesis, significantly differed between ezetimibe-treated cells and the nil group. RNA-related catalytic activity significantly differed between ezetimibe-treated and -untreated cells under Th17 differentiation conditions. However, rosuvastatin did not affect the expression levels of these genes. Hence, ezetimibe inhibits differentiation into Th17 cells and alters the functions of immune cells by modulating RNA processing.

RNA-Seq identified 15 genes based on KEGG pathways (IL1R1, IL17F, IL23R, IL6RA, IL6ST, JAK3, AHR, STAT3, HIF1α, RORC, IFNgR1, CD3G, CD3D, JUN, and FOXP3). These genes—except Foxp3—promote Th17 cell differentiation. Ezetimibe significantly reduced the expression levels of 14 of the Th17 differentiation related genes, except Foxp3. However, rosuvastatin significantly decreased the expression levels of only eight genes. Foxp3, which has anti-inflammatory effect, was significantly increased by both ezetimibe and rosuvastatin.

Our findings suggest that ezetimibe is useful for AS patients. When PBMCs from AS patients were treated with ezetimibe for 1 day *in vitro*, there was no significant difference in the Treg population, but the number of Th17 cells was significantly decreased compared to the untreated group. The levels of the proinflammatory cytokines IL-6, IL-17, and TNF-α were measured by ELISA in PBMC culture supernatant; these levels were significantly decreased by ezetimibe. Ezetimibe significantly reduced the number of Th17 cells among PBMCs from AS patients. However, mechanistic studies and clinical trials are needed.

Taken together, our findings showed that ezetimibe ameliorates the pathogenesis of AS by inhibiting Th17 cell activity and RNA processing. Therefore, ezetimibe has an independent anti-inflammatory effect on autoimmune disease. We confirmed the anti-inflammatory effect of ezetimibe *in vivo* in humans and mice, as well as *in vitro*. Statins typically have mild side effects but can cause severe muscle damage, inflammation of the liver and pancreas, and memory problems; they can also reduce the blood platelet count (59). Ezetimibe has fewer side effects than statins (60, 61). Although it is unclear whether a decrease in blood lipids induces AS remission, ezetimibe inhibits Th17 differentiation by decreasing blood lipid levels and inhibiting IL-23 receptor activity. Hence, ezetimibe has therapeutic potential for AS.

## Data availability statement

The data presented in the study are deposited in the NCBI Gene Expression Omnibus (GEO) repository, accession number GSE205850. The patients’ data are not publicly available due to ethical issues. Requests to access the datasets should be directed to Mi-La Cho, https://iammila@catholic.ac.kr.

## Ethics statement

The study protocol involving human participants was approved by the Institutional Review Board of Seoul St. Mary’s Hospital (KC17TNSI0237). The patients/participants provided their written informed consent to participate in this study. The experiments were assessed and approved by the Institutional Animal Care and Use Committee of the School of Medicine and the Animal Research Ethics Committee of the Catholic University of Korea (CUCM-2015-0063-01).

## Author contributions

JM, S-YL, HN, S-HP, and M-LC designed the experiments, analyzed the data and wrote the manuscript. The data of in vitro and in vivo experiments were performed by JM and AL. K-HC and JC conducted immunohistochemistry experiments. All authors critically reviewed and approved the final form of the manuscript.

## Funding

This research was supported by a grant of the Korea Health Technology R&D Project through the Korea Health Industry Development Institute (KHIDI), funded by the Ministry of Health & Welfare, Republic of Korea (grant number HI20C1496) and Basic Science Research Program through the National Research Foundation of Korea (NRF) funded by the Ministry of Education (grant number 2021R1I1A1A01056024).

## Conflict of interest

The authors declare that the research was conducted in the absence of any commercial or financial relationships that could be construed as a potential conflict of interest.

## Publisher’s note

All claims expressed in this article are solely those of the authors and do not necessarily represent those of their affiliated organizations, or those of the publisher, the editors and the reviewers. Any product that may be evaluated in this article, or claim that may be made by its manufacturer, is not guaranteed or endorsed by the publisher.
